# Proximal genomic localization of STAT1 binding and regulated transcriptional activity

**DOI:** 10.1186/1471-2164-7-254

**Published:** 2006-10-11

**Authors:** Samuel Wormald, Douglas J Hilton, Gordon K Smyth, Terence P Speed

**Affiliations:** 1 Division of Bioinformatics, The Walter and Eliza Hall Institute of Medical Research, Melbourne, Australia; 2 Division of Molecular Medicine, The Walter and Eliza Hall Institute of Medical Research, Melbourne, Australia; 3 Department of Statistics, University of California, Berkeley, California, USA

## Abstract

**Background:**

Signal transducer and activator of transcription (STAT) proteins are key regulators of gene expression in response to the interferon (IFN) family of anti-viral and anti-microbial cytokines. We have examined the genomic relationship between STAT1 binding and regulated transcription using multiple tiling microarray and chromatin immunoprecipitation microarray (ChIP-chip) experiments from public repositories.

**Results:**

In response to IFN-γ, STAT1 bound proximally to regions of the genome that exhibit regulated transcriptional activity. This finding was consistent between different tiling microarray platforms, and between different measures of transcriptional activity, including differential binding of RNA polymerase II, and differential mRNA transcription. Re-analysis of tiling microarray data from a recent study of IFN-γ-induced STAT1 ChIP-chip and mRNA expression revealed that STAT1 binding is tightly associated with localized mRNA transcription in response to IFN-γ. Close relationships were also apparent between STAT1 binding, STAT2 binding, and mRNA transcription in response to IFN-α. Furthermore, we found that sites of STAT1 binding within the Encyclopedia of DNA Elements (ENCODE) region are precisely correlated with sites of either enhanced or diminished binding by the RNA polymerase II complex.

**Conclusion:**

Together, our results indicate that STAT1 binds proximally to regions of the genome that exhibit regulated transcriptional activity. This finding establishes a generalized basis for the positioning of STAT1 binding sites within the genome, and supports a role for STAT1 in the direct recruitment of the RNA polymerase II complex to the promoters of IFN-γ-responsive genes.

## Background

Interferon-gamma (IFN-γ) is a potent pro-inflammatory cytokine that regulates a spectrum of biological processes, and is produced primarily in response to infection [1]. IFN-γ signal transduction begins at the cell surface with the formation of a heteromeric protein complex that includes IFN-γ, IFN-γ receptor-1, and IFN-γ receptor-2 [1]. Associated with the IFN-γ receptors are members of the Janus kinase (JAK) family of tyrosine kinases, which become activated upon formation of the IFN-γ receptor complex, and in turn phosphorylate the signal transducer and activator of transcription-1 (STAT1) transcription factor [2-4]. Upon its phosphorylation, STAT1 homo-dimerizes, and is transported into the nucleus where it binds to the gamma activated sequence (GAS; consensus: TTCNNNGAA [5]) to activate the expression of IFN-γ-responsive genes [6]. One example of a STAT1-responsive gene is the interferon regulatory factor-1 (IRF1) gene, an important IFN-γ-responsive transcription factor which contains a functional GAS 120 bp upstream of its first exon [7,8]. In addition, STAT1 functions as a component of the IFN stimulated gene factor 3 (ISGF3) transcription factor complex, which also includes STAT2 and interferon regulatory factor-9 (IRF9) [9,10]. The ISGF3 complex is formed in response to signaling by IFN family members, including IFN-α, that associate with IFN-α receptor-1 and IFN-α receptor-2. Upon its transportation into the nucleus, ISGF3 binds to IFN-stimulated response elements (ISREs; consensus: GGAAANNGAAACT [11]) to activate the expression of IFN-α-responsive genes.

How gene expression is regulated by the association of transcription factors to their target sequences is a central question in mammalian biology. Compared with lower organisms such as *S. cerevisiae *and *D. melanogaster*, the genomic regions responsible for regulating the expression of mammalian genes are much more difficult to predict, and may be located far away from a gene's transcriptional start site (TSS). Furthermore, the debate over what constitutes a gene was further intensified with the development of tiling arrays for the human genome, and the discovery that much of the human transcriptome is coded for by regions of the genome that lie outside of exons as they have been classically defined [12,13]. Chromatin immunoprecipitation microarray (ChIP-chip) technology provided additional insight into the regulation of the human transcriptome when it was employed to examine the transcription factor binding sites (TFBSs) of Sp1, cMyc, and p53 on chromosomes 21 and 22 [14]. Interestingly, only 22% of these TFBSs were located in the upstream regions of genes, the regions that have classically been defined as "promoter" regions. The genomic relationship between sites of transcription factor binding, and sites of transcription, was further elucidated in an elegant study of estrogen receptor (ER) binding across chromosomes 21 and 22 [15]. Remarkably, the RNA polymerase II (RNApolII) complex was found to associate in an estrogen-dependent manner with the majority of tested TFBSs, even those located far from the nearest TSS. Using a chromatin capture assay, an ER binding site located over 144 kbp from the NRIP-1 gene was shown to function as an enhancer of NRIP-1 transcription. These results showed that, when bound to its TFBS, the ER can act as an enhancer to regulate the expression of target genes by associating, often across large chromosomal distances, with RNApolII.

Recently, the response to IFN-γ has been the subject of microarray expression analyses as well as ChIP-chip analyses for STAT1 [16-20]. Hartman *et al*. compared the locations of STAT1 binding sites on chromosome 22 (as determined by ChIP-chip) with the expression level of the nearest gene, and noted that only 21% of STAT1 binding sites were within 10 kbp of the start of the nearest gene. Hartman *et al*. also suggested that a novel mechanism may alter the specificity of STAT1 binding, depending on whether it is induced by IFN-α or IFN-γ.

Elucidating the rationale with which transcription factor binding sites are deployed across the genome will likely be of great benefit to our understanding of the complexities of transcriptional regulation in mammals. We have examined the data associated with several recent tiling array experiments, and show here that STAT binding and mRNA transcription in response to IFN are much more closely associated than previous reports would suggest. Our results indicate that, in contrast to other mammalian transcription factors such as the ER, STAT1 binds closely to the TSSs of its target genes. Our findings also suggest that STAT1 may play an important role in recruiting the RNApolII complex to the promoters of IFN-γ-inducible genes.

## Results

### STAT1 binding and mRNA transcription in response to IFN-γ are correlated at the probe level for chromosome 22 tiling arrays

**Figure 1:**
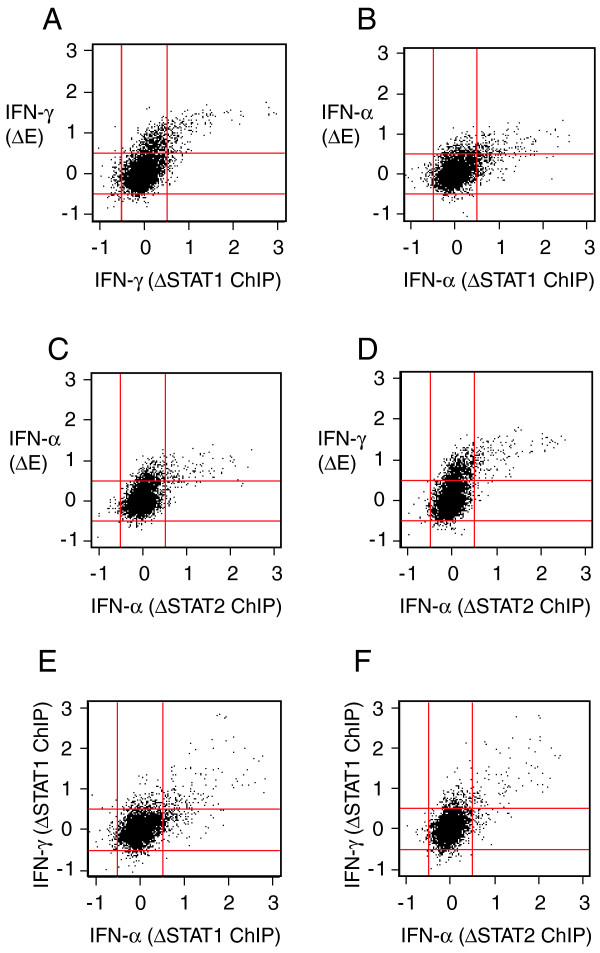
**Probe level relationships between STAT binding and mRNA transcription in response to IFN**. Differences in probe intensities for chromosome 22 arrays are plotted for the responses of HeLaS3 cells stimulated with IFN-γ or IFN-α (relative to non-stimulated controls). ΔSTAT = differential STAT binding; ΔE = differential expression. (A) IFN-γ (ΔE) vs IFN-γ (ΔSTAT1). (B) IFN-α (ΔE) vs IFN-α (ΔSTAT1). (C) IFN-α (ΔE) vs IFN-α (ΔSTAT2). (D) IFN-γ (ΔE) vs IFN-α (ΔSTAT2). (E) IFN-γ (ΔSTAT1) vs IFN-α (ΔSTAT1). (F) IFN-γ (ΔSTAT1) vs IFN-α (ΔSTAT2). All raw data were obtained from Hartman *et al*.'s recent analysis of transcription and STAT binding in response to IFN [16]. All values are on the log_2 _scale. Red lines indicate cutoffs of ± 0.5 (on the log_2 _scale).

To examine the relationship between STAT1 binding and mRNA transcription in response to IFN-γ, we obtained the data associated with Hartman *et al*.'s tiling array analysis of gene transcription and STAT1 binding for chromosome 22 following treatment with IFN-γ and IFN-α [16]. We were particularly interested in determining whether there was any evidence for localized coupling of differential STAT1 binding and differential mRNA expression. To our surprise, there was a pronounced probe level correlation between differential STAT1 binding in response to IFN-γ, and differential mRNA expression in response to IFN-γ (Figure 1A). A similar probe level relationship between STAT1 binding, STAT2 binding, and differential expression, was also apparent for the responses of cells to IFN-α (Figure 1B,C). Furthermore, probes that detected differential STAT2 binding in response to IFN-α also detected substantial differential expression in response to IFN-γ (Figure 1D), and consistent with this, there was a high probe level correlation between the binding of STAT1 or STAT2 in response to IFN-α, and the binding of STAT1 in response to IFN-γ (Figure 1E,F). Consistent with Hartman *et al*.'s conclusion that the binding preference of STAT1 may differ in response to IFN-α versus IFN-γ [16], some differences in the binding sites of STAT1 were apparent for these stimuli (Figure 1E), although this was primarily true only of some of the relatively weak STAT1 binding sites. For relatively strong STAT1 binding sites, the binding preferences in response to IFN-γ versus IFN-α showed substantial correlation (Figure 1E).

The findings outlined above strongly suggest that there is a high degree of overlap between the positions where STAT1 and STAT2 bind in response to either IFN-γ or IFN-α, which is perhaps not surprising, since STAT2 binds DNA as part of the ISGF3 transcription factor complex, of which STAT1 is also a component [10]. Consistent with this, correlation was also apparent between the transcriptional response of cells to IFN-γ and the transcriptional response of cells to IFN-α (data not shown). Of greater interest, however, is the clear probe level correlation between the binding of STAT1 or STAT2, and the differential expression in response to IFN-γ or IFN-α. Given that probes for the chromosome 22 arrays are 300 – 1400 bp long [13], our observations indicate that the locations at which STAT1 or STAT2 associate with chromosome 22 are in the majority of cases very close to regions of chromosome 22 that are transcriptionally regulated by IFN-γ or IFN-α.

### Detection of STAT1 binding sites within the ENCODE region

**Figure 3:**
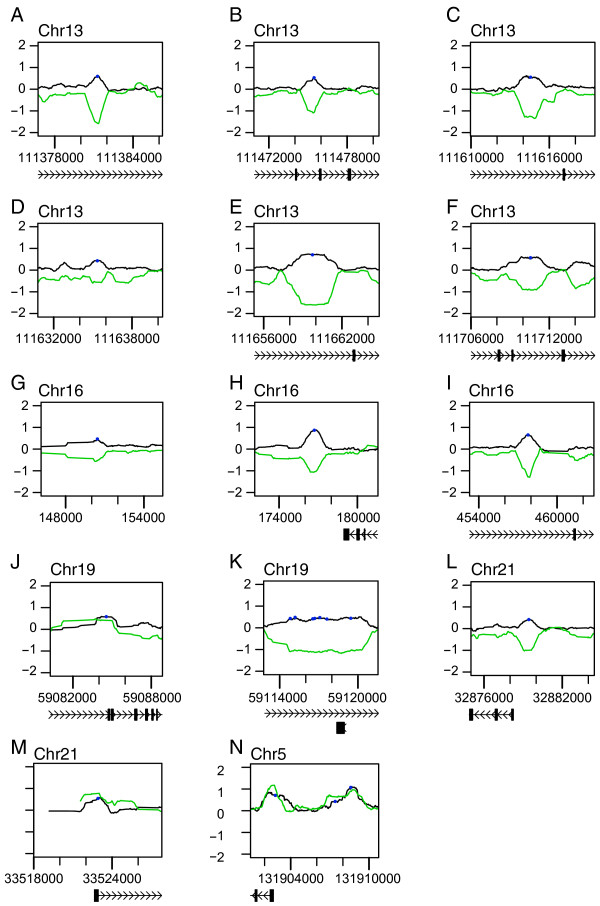
**STAT1 binding in response to IFN-γ is positively and negatively correlated with RNApolII binding**. (A – N) Levels of differential STAT1 binding in response to IFN-γ (ΔSTAT1; black line) are plotted for locations within the ENCODE region where STAT1 binding sites (blue circles) were identified. Levels of RNApolII binding in non-stimulated HeLaS3 cells are shown in green ("0" corresponds to average RNApolII binding across the ENCODE region). Black boxes indicate the locations of exons; connecting lines indicate intronic regions; arrowheads within introns indicate the direction of transcription. Raw data were obtained from Rozowsky *et al'*s tiling array analysis of STAT1 binding in response to IFN-γ [18], and Bieda *et al*.'s tiling array of RNApolII binding in non-stimulated cells [25].

To examine STAT1 binding in response to IFN-γ at a higher resolution than is permitted by cDNA microarray technology, we obtained data associated with a recent ChIP-chip analysis of STAT1 binding in response to IFN-γ [18] across the 1 Mb encyclopedia of DNA elements (ENCODE) region [21]. A moderately conservative differential intensity cut-off of 2^0.4 ^(1.3-fold) was selected by inspection of a quantile-quantile plot of the differential intensities (data not shown). In total, 463 probes satisfied this cut-off. These probes all clustered to 22 distinct genomic sites, 17 (77%) of which were within 10 kbp of the nearest 1st exon of a gene (Figure 3 and Table 1). This method appears to identify fewer STAT1 binding sites than are identified on the UCSC genome browser [22], presumably because our significance cut-off is higher. When the alternative transcripts of three genes, ATP11A, MCF2L, and RAB11-FIP3, were accounted for, 21 of the 22 (95%) of the STAT1 binding sites we identified were within 10 kbp of the nearest 1^st ^exon of a gene (Table 1). Of the 11 genes identified in this manner, three (IRF1, PRKCG, and F7) are known IFN-γ-responsive genes [7,8,23,24]. In the case of IRF-1, a STAT1 binding site immediately upstream of the first exon has been previously identified by *in vivo *footprinting [7].

### The majority of STAT1 binding sites exhibit localized suppression of RNApolII binding in non-stimulated cells

Our finding that IFN-γ-induced STAT1 binding to chromosome 22 is closely associated with increased mRNA transcription suggested that STAT1 may bind proximally to RNApolII within the promoters of IFN-γ-responsive genes. We were therefore interested in examining the binding of RNApolII to regions near STAT1 binding sites. To this end, we obtained data associated with Bieda *et al*.'s recent analysis of RNApolII binding within the ENCODE region for non-stimulated HeLaS3 cells [25]. Remarkably, the binding of RNApolII in non-stimulated cells was negatively correlated (on the log_2 _scale) with the binding of STAT1 in response to IFN-γ for 17 of the 22 binding sites within the ENCODE region (Figure 3A–I, K, L). This is indicative of a diminished level of RNApolII binding, relative to the average level of RNApolII binding for the entire ENCODE region. Localized suppression of RNApolII binding is likely to reflect the locations at which modifications in the chromatin state, or possibly other factors, prevent the association of RNApolII (relative to the average level of association across the ENCODE region).

### RNApolII binds to STAT1 binding sites near the IRF1 and IFNaR1 genes in non-stimulated cells

Of the five STAT1 binding sites that did not exhibit suppressed RNApolII binding in non-stimulated cells (Figure 3J, M, N), three were located within 7 kbp upstream of the IRF1 gene (Figure 3N), and one was located near the start of the IFNaR1 gene (Figure 3M). In each case, STAT1 binding in response to IFN-γ was positively correlated with RNApolII binding, relative to the average level of RNApolII binding across the ENCODE region.

### Binding of RNApolII and localized mRNA transcription are associated with STAT1 binding sites near the IRF1 and IFNaR1 genes in non-stimulated cells

**Figure 4:**
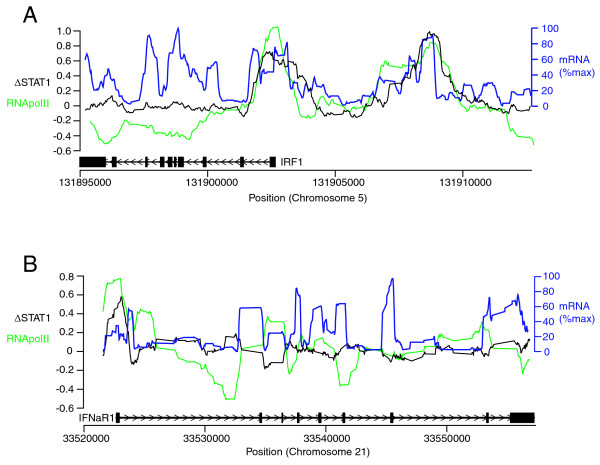
**Detection of basal mRNA transcription near binding sites for STAT1 and RNApolII**. The binding of STAT1 in response to IFN-γ was compared with the binding of RNApolII and the transcription of mRNA in non-stimulated cells. Black line (ΔSTAT1): log_2 _of differential STAT1 binding in cells stimulated with IFN-γ. Green line (ΔRNApolII): log_2 _of differential RNApolII binding, relative to the average binding of RNApolII across the ENCODE region, in non-stimulated cells. Blue line (mRNA %max): percentage of maximum mRNA detection score across the window. Black boxes indicate the locations of exons; connecting lines indicate intronic regions; arrowheads within introns indicate the direction of transcription. (A) IRF1 gene and upstream region. (B) IFNaR1 gene. Raw data were obtained from Rozowsky *et al'*s tiling array analysis of STAT1 binding in response to IFN-γ [18], Bieda *et al*.'s tiling array of RNApolII binding in non-stimulated cells [25], and Sekinger et al.'s tiling array analysis of polyA RNA levels in non-stimulated cells [37].

Our finding that IFN-γ-induced STAT1 binding to chromosome 22 is closely associated with increased mRNA transcription indicated that STAT1 binds closely to transcriptionally-active regions of the genome. Our subsequent identification of STAT1 binding sites that are bound by RNApolII near the start positions of the IRF1 and IFNaR1 genes in non-stimulated cells suggested that basal levels of mRNA transcription might be detectable at these sites. To investigate this possibility, we obtained data associated with a recent analysis of basal mRNA transcription in non-stimulated HeLa cells for 10 human chromosomes [26]. Pleasingly, most exons of the IRF1 and IFNaR1 genes exhibited clear peaks of basal mRNA detection (Figure 4A, B). Basal mRNA transcription was apparent not only within exons, but also introns and extragenic regions (Figure 4A, B). All peaks of STAT1 binding near the IRF1 and IFNaR1 genes overlapped with peaks of basal mRNA transcription as well as peaks of basal RNApolII binding (Figure 4A, B), thus reinforcing our finding using Hartman *et al*.'s chromosome 22 data [16] that STAT1 binding to chromosome 22 is closely associated with increased mRNA transcription in response to IFN-γ.

### STAT1 binds proximally to the start positions of genes with key roles in the cellular response to IFN-γ

When conducting microarray analyses of cellular responses to stimuli, it is often difficult to distinguish genes that are obligatory components of the cell's response from genes that are induced only in particular cell types or under certain conditions, and may therefore be less critical to the outcome of the response. It is interesting to note that within the ENCODE region, STAT1 bound most closely to start positions of two genes with key roles in IFN signal transduction, IRF1 and IFNaR2 (Table 1). In fact, these are the only genes for which proximal STAT1 binding in the ENCODE region was observed that have known roles in IFN signal transduction. We wondered therefore whether STAT1 binding sites are also closely associated with the start positions of other "key" IFN-γ-inducible genes. To this end, we identified 10 genes for which the transcriptional response to IFN-γ is conserved between human microglial cells and mouse livers, and are thus likely to be essential components of the responses of most cells to IFN-γ (Table 2). Remarkably, the expression levels of seven of these genes are known to be regulated by STAT1 binding sites located within just 200 bp of the start of each gene (Table 2). This close proximity with which STAT1 regulates the expression of key IFN-γ-responsive genes is in stark contrast to the relatively large distances between most STAT1 binding sites within the ENCODE region, and the start positions of the nearest genes (Table 1). This suggests that genes with STAT1 binding sites that are not located close to the start of the coding region could be more susceptible to tissue-specific mechanisms of regulation, for example by chromatin modification of the promoter.

## Discussion

Whilst it is clear that mammalian promoter regions are often enriched for TFBSs, much remains to be learnt about how mammalian TFBSs are distributed throughout the genome, and what determines the positioning of TFBSs. We analyzed data from Hartman *et al*.'s study of IFN-induced STAT binding and mRNA transcription for chromosome 22 [16], and to our surprise, we observed a marked probe level correlation between STAT1 binding and differential expression in response to IFN-γ. Although all ChIP-chip analyses of transcription factor binding have the potential to detect some RNApolII binding sites due to interactions between RNApolII and the transcription factor of interest, our results suggest that the vast majority of STAT1 binding sites, if not all STAT1 binding sites, are proximal to transcriptionally active regions. The extent to which we observed this phenomenon indicates that STAT1 binding sites are located proximally to regions that are bound by the transcriptional apparatus.

In addition to the discovery of a probe level correlation between mRNA transcription and STAT1 binding in response to IFN-γ, our analysis of Hartman *et al*.'s Chromosome 22 tiling array data for STAT binding and mRNA transcription in response to IFN [16] revealed probe level correlations for various combinations of STAT1 binding, STAT2 binding, and mRNA transcription in response to treatment with either IFN-γ or IFN-α. Together, these observations suggest that differences in the binding specificities between STAT1 and STAT2 are predominantly quantitative rather than qualitative. Consistent with this idea, IFN-α and IFN-γ were recently shown to induce the transcription of similar sets of genes [20]. The substantial correlation we observed between the binding sites of STAT1 in response to IFN-α vs IFN-γ perhaps casts some doubt over the significance of Hartman *et al*.'s observation that the binding preference of STAT1 is differentially regulated in response to IFN-α as compared to IFN-γ [16].

Consistent with our observation of a probe level correlation between STAT1 binding and mRNA expression in response to IFN-γ for Chromosome 22, we found that STAT1 binds specifically to sites within the ENCODE region that exhibit either diminished or enhanced binding of RNApolII in non-stimulated cells. Collectively, these findings suggest that STAT1 binds proximally to regions of the genome that are involved in the initiation of RNA transcription, since this would explain both the correlation with mRNA transcription that we identified using the Chromosome 22 tiling array data, and the correlation with differential RNA polymerase binding that we identified using the ENCODE tiling array data. The primary mechanism by which STAT1 activates gene transcription may therefore be to recruit RNA polymerase II to the promoters of IFN-γ-responsive genes.

There have been several reports implicating STAT1 in the recruitment of components of the transcriptional apparatus to the promoters of particular IFN-responsive genes. In 2003, STAT1 was shown to synergize with NF-κB at the promoter of the CXCL9 gene to bind the transcriptional co-activator, CREB-binding protein, which may in turn recruit the RNApolII complex [27]. In 2004, it was shown that IRF9 (a member of the ISGF3 complex) may recruit the RNApolII complex to the promoter of interferon-stimulated gene 54 in a histone deacetylase-dependent manner [28]. In 2005, STAT1 was shown to recruit the DNA helicase protein mini-chromosome maintenance-5 to the promoters of the genes encoding IRF1, TAP1, guanylate-binding protein-1, and class II transactivator in response to IFN-γ [29]. Furthermore, transcription of the aforementioned genes in response to IFN-γ was dependent on the activity of the mini-chromosome maintenance-5 protein, and the binding of the mini-chromosome maintenance-5 protein to the IRF1 gene was consistent with the binding of RNApolII to the IRF1 gene [29]. Our findings that STAT1 binds to positions on chromosome 22 that exhibit IFN-γ-inducible mRNA transcription, and to positions within the ENCODE region that exhibit either enhanced or diminished RNApolII binding (relative to the average level of RNApolII binding), are in strong agreement with the above reports. Furthermore, our results generalize the findings of the previous reports, since they suggest that recruitment of the RNApolII complex to the promoters of IFN-γ-responsive genes is the dominant mechanism by which STAT1 activates gene transcription. At STAT1 binding sites that are normally inaccessible to RNApolII, this could involve chromatin modification to make the site more accessible to RNApolII. At STAT1 binding sites that exhibit basal levels of RNApolII binding, STAT1 may simply enhance RNApolII binding. Such a mechanism of transcriptional regulation contrasts with that of the ER, which can regulate the transcription of genes from distant ER TFBSs by acting as an enhancer for RNApolII activity [15].

**Figure 5:**
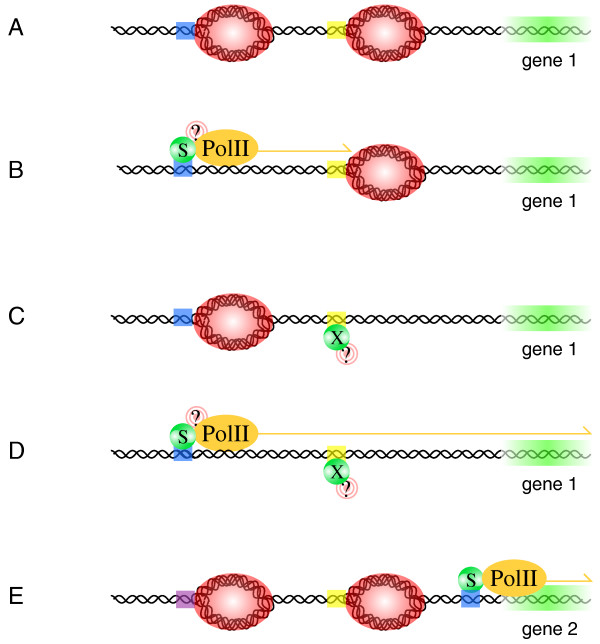
**A hypothetical mechanism by which condition-specific or non-specific transcriptional responses to IFN-γ may be achieved**. (A – D) Condition-specific responsiveness to IFN-γ of a gene, "gene 1". Condition A: STAT1 ("S") and transcription factor-X ("X") are inactive, and hence the binding sites for STAT1 (blue DNA) and X (yellow DNA) are unoccupied. In this state, chromatin-modifying proteins render regions near the binding sites for STAT1 and X (red DNA) inaccessible to RNApolII. Condition B: In this condition, STAT1 is activated by IFN-γ, and associates with its binding site upstream of gene 1. STAT1 recruits chromatin-modifying proteins ("?") to make the region accessible to RNApolII; STAT1 then recruits RNApolII to DNA near the STAT1 binding site. RNApolII is unable to transcribe gene 1, however, because the chromatin configuration near the binding site for X inhibits its progression. Condition C: In this condition, X is active, and associates with its binding site upstream of gene1 to recruit chromatin-modifying proteins, which make the region accessible to RNApolII. However, RNApolII is not actively recruited to the gene 1 promoter, so gene 1 is not transcribed. Condition D: In this condition, both STAT1 and X associate with their binding sites, and recruit chromatin-modifying proteins to make the promoter accessible to RNApolII. STAT1 then recruits RNApolII, and RNApolII is able to progress along the promoter to transcribe gene 1. (E) Non-specific transcriptional responsiveness to IFN-γ of a gene, "gene 2". Gene 2 is regulated by transcription factors that associate with binding sites (blue, yellow and purple DNA) within its promoter. Gene 2 has a key role in regulating the response to IFN-γ, so it is important that it is induced by IFN-γ in all tissues, under all conditions. The STAT1 binding site is therefore located close to the start of gene 2, where other regulatory elements within the promoter will not affect the ability of STAT1 to induce transcription of gene 2.

Interestingly, two STAT1 binding sites upstream of two well characterized mediators of IFN signal transduction, IFNaR1 and IRF1, were the closest two STAT1 binding sites to the start of any gene in the ENCODE region (both were located less than 500 bp away). This could simply be a coincidence, however our identification of ten genes for which transcription in response to IFN-γ is conserved between different tissues of humans and mice provides an interesting alternative explanation. Whilst many genes are probably induced by IFN-γ only in certain cell types or under certain conditions, these ubiquitously-responsive genes are likely to play key roles in the response to IFN-γ of numerous cell types (just as IFNaR1 and IRF1 do). Consistent with this hypothesis, genes with important functions in cytokine signaling were identified, including SOCS-1 and -3 [30], IRF1 [8], and the chemokines CXCL9 and CXCL10 [31]. Remarkably, seven of the ten ubiquitously-IFN-γ-inducible genes that we identified are known to be regulated by STAT1 binding sites that are located within 200 bp from the start of each gene. Of the remaining three, little is known about the roles of GADD45γ and ID2 in response to IFN-γ, and SOCS-1 may be regulated primarily by IRF1, and not STAT1 [19]. The close proximity with which STAT1 regulates the expression of "core" IFN-γ-inducible genes suggests that many of the STAT1 binding sites identified by ChIP-chip may be involved in the regulation of genes, alternative gene transcripts, or non-coding RNAs that are induced by IFN-γ only in certain cell types or only under certain conditions. As illustrated in Figure 5, increased regulatory opportunities may be available for genes that are regulated by STAT1 from a distance, compared with genes that are regulated by STAT1 close to the start of the coding region. This model contrasts with a recent report showing that different combinations of transcription factor binding sites in the proximal promoter region are important for determining tissue-specific gene expression [32]. However, given that biological systems are inherently variable, it seems unlikely that the two models are necessarily mutually exclusive.

## Conclusion

Our analysis of STAT1 binding and mRNA transcription reveals that the binding of STAT1 is closely associated with IFN-γ-inducible transcriptional activity. Furthermore, our findings implicate STAT1 in the regulation of RNApolII binding, and suggest that recruitment of the RNApolII complex to the promoters of IFN-γ-responsive genes could be a general mechanism by which STAT1 activates transcription. Finally, we propose that STAT1 may strategically associate with the start positions of key IFN-γ-responsive genes to ensure that they are ubiquitously, rather than conditionally, induced in response to IFN-γ.

## Methods

### Analysis of chromosome 22 cDNA tiling array data

Genepix files corresponding to Hartman *et al*.'s [16] analysis of STAT binding and mRNA transcription in response to 120 min of IFN stimulation were obtained on-line [33]. All experiments were performed using HeLaS3 cells. These cDNA tiling arrays are designed to detect chromosome 22 DNA using 300 – 1400 bp probes, which are aligned with chromosome 22 in an end-to-end configuration [13]. The complete set of chromosome 22 cDNA tiling arrays is comprised of three different arrays, numbered 1 to 3 [13]. In all cases where plots of data from chromosome 22 arrays are presented here, the data correspond to array-2 of the three-array set, and are representative of arrays -1 and -3. Data from 4 – 6 replicates were analyzed for all treatments. We processed and analyzed Genepix data using the limma package [34] for the R statistical programming environment, as follows. Background correction was performed using limma's "normexp" method. Within-array normalization was achieved using print-tip loess normalization. Between-array normalization was achieved using quantile normalization of mean probe intensity values, which is implemented by limma as the "Aquantile" method. Correlations in probe intensity levels ("probe level correlations") between different IFN treatments, and between either mRNA transcription or chromatin immunoprecipitation for STAT1, were visually assessed using scatterplots. Probe intensity levels for IFN-stimulated cells showed no correlation with NF-kappaB binding in response to tumor necrosis factor-alpha [35], indicating that the correlations observed are specific to the response to IFN (data not shown).

### Analysis of ENCODE oligonucleotide tiling array data

Cy5 and Cy3 probe intensity values for Rozowsky *et al*.'s [18] 36-base oligonucleotide tiling array analysis of STAT1 binding in HeLaS3 cells in response to 30 min of IFN-γ stimulation were obtained from the Gene Expression Omnibus (GEO) website [36], series id GSE2714. These data are also available as a track on the UCSC genome browser [22]. Experiments were performed in duplicate. These tiling arrays are designed to detect the 30 Mb Encyclopedia of DNA Elements (ENCODE) region [21]. Probes are 36 bases in length, and are aligned with DNA from the ENCODE region in an end-to-end configuration. We used limma [34] to perform within-array normalization (using the "loess" method) and quantile normalization of mean probe intensity values (using the "Aquantile" method). To enhance the identification of runs of probes that detect STAT1 binding, probe intensities were smoothed by taking the mean intensites across a window of 20 probes (720 bp).

Cy5 and Cy3 probe intensity values for Bieda *et al*.'s [25] 50-base oligonucleotide tiling array analysis of RNApolII binding in non-stimulated HeLaS3 cells were obtained from the Gene Expression Omnibus (GEO) website [36], series id GSE4337. Experiments were performed in triplicate. These tiling arrays are designed to detect the ENCODE region [21]. Probes are 50 bases in length, and are aligned with DNA from the ENCODE region in a 12-base overlap configuration. We used limma [34] to perform between-array normalization (using the "loess" method) and quantile normalization of mean probe intensity values (using the "Aquantile" method). To enhance the identification of runs of probes that detect RNApolII binding, probe intensities were smoothed by taking the mean intensites across a window of 20 probes (760 bp). To perform probe level comparisons between these 50-base oligonucleotide arrays, and the 36-base oligonucleotide arrays for Rozowsky *et al*.'s [18] ChIP-chip analysis of STAT1 binding within the ENCODE region, probe intensities from the 50-base overlapping format were mapped to the 36-base end-to-end format. Weighted averages were calculated where two 50-base probes overlapped with a single 36-base probe.

Polyadenylated (polyA) RNA signal values for Sekinger *et al*.'s [37] tiling array analysis of non-stimulated HeLaS3 cells were obtained from the GEO website, series id GSE2800. The tiling arrays used for these experiments were manufactured by Affymetrix (Santa Clara, CA), and have 25 base probes that detect DNA from the ENCODE region in a 5-base-overlap configuration. Signal values were determined using the Wilcoxon Sign Rank Scan Statistic, as outlined in the description accompanying each sample. To enhance the identification of runs of probes that detect the presence of polyA RNA, we smoothed probe intensity values by taking the mean intensity across a window of 20 probes (400 bp).

### Analysis of Affymetrix GeneChip expression data

Probe intensity values for Rock *et al*.'s microarray analysis of the transcriptional response of human fetal microglial cells to 1 h of IFN-γ treatment [17] were obtained from the GEO website, series id GSE1432. All experiments were performed in quadruplicate. Affymetrix GeneChip HGU133A arrays, which detect the expression of approximately 16,000 human genes, were used for this experiment. We used limma [34] to perform within-array (loess) and between-array (quantile) normalization of probe intensity data, and to identify significantly differentially expressed genes.

Affymetrix MGU74Av2 CEL files for our previously reported timecourse analysis of the transcriptional response of IFN-γ^-/- ^mouse livers to intraperitoneal injection with IFN-γ [19] (GEO series id GSE4232) were processed by the affy package [38-40] for the R statistical programming environment [41]. Background correction, quantile normalization, and expression summaries were performed using affy's "robust multiarray average" method, with default parameters. Samples corresponding to 0 h, 0.5 h, 1 h, 2 h, 4 h, 12 h, 16 h, 24 h and 48 h after stimulation with IFN-γ were included in the analysis.

### Data analysis

All analyses of data were carried out using the R statistical programming environment[41]. Levels of differential expression were determined by limma [34] using empirical Bayes statistics [42].

## Authors' contributions

SW designed the study, performed data analysis, interpreted results, and wrote the manuscript. GKS contributed to data analysis, interpretation of results, and assisted with writing the manuscript. DJH and TPS contributed to the design of the study, interpretation of results, and revision of the manuscript.

## Figures and Tables

**Figure 2:**
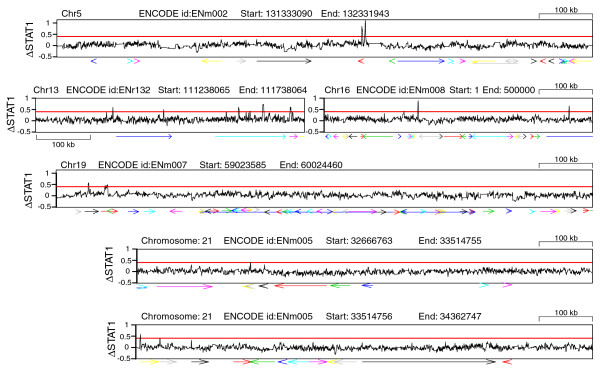
**Distribution of STAT1 binding sites within the ENCODE region**. Levels of differential STAT1 binding in response to IFN-γ (ΔSTAT1; black line) are plotted for ENCODE regions containing one or more probes exhibiting differential binding over a cut-off of 0.4 (red line). Probe intensities were smoothed as described in Methods. Arrows indicate locations and directions of known genes, and are colored to improve visual discrimination. All raw data were obtained from Rozowsky *et al*.'s tiling array analysis of STAT1 binding in response to IFN-γ [18]. Differential STAT1 binding values are on the log_2 _scale.

**Table 1:** Distribution of STAT1 binding sites detected within the ENCODE region.

**Chr**	**Position**	**Distance**	**Gene symbol**	**Description**	**RefSeq id**	**Figure**
13	111381278	-6172	ATP11A*	ATPase I type 11A	BC024154	3A
	111475471	+88021 (+1162)			BC024154 (AK024264)	3B
13	111614528	+43712 (+9528)	MCF2L*	MCF.2-like	NM_024979 (BC020208)	3C
	111635357	+64541 (+30357)				3D
13	111659869	+48237 (-6750)	F7 (MCF2L*)	Coagulation factor VII precursor	NM_000131 (AB116075)	3E
	111710547	+2441	F7		NM_000131	3F
16	150458	-5539	HBM	Hemoglobin mu chain (Hypothetical protein)	NM_001003938	3G
16	176687	+6352	HBQ1	Hemoglobin theta-1 chain	NM_005331	3H
16	457805	+42185 (-3412)	Rab11-FIP3*	Rab11 family-interacting protein 3	NM_014700 (BC009036)	3I
19	59084649	+7247	PRKCG	Protein kinase C, gamma	NM_002739	3J
19	59114793	+4154	-	Ensembl predicted	-	3K
	59115225	+3722		gene		
	59116521	+2426		ENSG00000188966		
	59116611	+2336				
	59117115	+1832				
	59117619	+1328				
	59119491	-544				
21	32879469	-1229	TCP10L	t-complex 10 (mouse)-like	NM_144659	3L
21	33523051	+376	IFNaR2	Interferon (alpha, beta and omega) receptor 2	NM_000874	3M
5	131902803	-160	IRF1	Interferon regulatory	NM_002198	3N
	131907429	-4786		factor-1		
	131908640	-5997				

The genomic positions at which STAT1 binds in response to IFN-γ were determined by taking the average position of each peak of STAT1 binding with a score greater than 0.4, as described in Figure 2. Distances from the start sites of nearest genes are given in base-pairs (according to NCBIv34 annotation). References to Figure 3, showing graphical illustrations of STAT1 binding intensities, are given for each STAT1 binding site.

**Table 2:** Genes induced by IFN-γ in both human and mouse tissues, and associated STAT1 binding sites.

**Gene Symbol**	**Description**	**STAT1 TFBS**	**Reference**
BCL6	B-cell leukemia/lymphoma-6	+192 bp	[43]
CXCL10	Chemokine (C-X-C motif) ligand-10	-153 bp	[44]
CXCL9	Chemokine (C-X-C motif) ligand-9	-167 bp	[45-47]
GADD45γ	Growth arrest and DNA-damage-inducible 45 gamma	unknown	
ID2	Inhibitor of DNA binding-2	unknown	
IRF1	Interferon regulatory factor-1	-120 bp	[7]
IFIT2	Interferon-induced protein with tetratricopeptide repeats-2	-85 bp	[48]
SOCS1	Suppressor of cytokine signalling-1	unknown	
SOCS3	Suppressor of cytokine signalling-3	-64 bp	[49]
TAP1	Transporter 1, ATP-binding cassette, sub-family B	+135 bp	[50]

Genes exhibiting significant (p < 0.01) differential expression in human microglial cells stimulated with IFN-γ for 1 h were identified by limma [34, 42] analysis of data associated with Rock *et al*.'s recent publication [17] (GEO id GSE1432). 80 orthologous mouse genes were identified using HCNetDat [51]. Differential expression levels in response to IFN-γ for the orthologous mouse genes were determined by affy [38-40] analysis of data associated with our recent publication of transcriptional responses to IFN-γ in IFN-γ^-/- ^mouse livers [19] (GEO id GSE4232). Literature was searched for experimentally verified STAT1 TFBSs (either GAS sites or ISRE sites were accepted) associated with the 10 most differentially regulated orthologous mouse genes (each was induced over 1.6-fold). Positions of STAT1 binding sites are given relative to the start of the first exon of each gene.
